# Probing local atomic structure in thin films by grazing-incidence total X-ray scattering with a convergent X-ray beam

**DOI:** 10.1107/S1600576726007181

**Published:** 2026-08-24

**Authors:** Masatsugu Yoshimoto, Kazuki Ito, Kazuhiko Omote

**Affiliations:** a X-ray Research Laboratory, Rigaku Corporation, Matsubara-cho 3-9-12, Akishima, Tokyo, Japan; University of Silesia in Katowice, Poland

**Keywords:** grazing-incidence total X-ray scattering, GI-TXS, convergent incident X-ray beams, thin film local structure, pair distribution functions

## Abstract

Grazing-incidence total X-ray scattering (GI-TXS) is an important technique for evaluating the short-range order and medium-range order structures of thin films. A high-quality pair distribution function requires high-intensity total scattering data over a wide scattering vector magnitude (*Q*) range, which necessitates the use of high-energy X-rays. However, a lower incident angle leads to a wider footprint at the sample position, resulting in a trade-off between achieving high-intensity total scattering and using high-energy X-rays in conventional laboratory setups. In this study, we performed GI-TXS measurements on an indium tin oxide (ITO) thin film using a convergent incident X-ray beam to maximize the X-ray flux at the sample position. By incorporating the convergence angle distribution and thin film absorption parameters, the substrate contribution to the observed total scattering intensity was quantitatively estimated and corrected. As a result, the total scattering profile of the ITO thin film was successfully obtained up to *Q* = 21 Å^−1^ using a laboratory X-ray diffractometer.

## Abstract 2

We performed grazing-incidence total X-ray scattering on an indium tin oxide thin film using a convergent incident beam. This approach enables the acquisition of total scattering profiles up to *Q* = 21 Å^−1^, demonstrating the feasibility of laboratory instruments for thin film pair distribution function analysis.

## 1. Introduction

Thin films are key materials used in a wide range of fields, including electronic devices, optical devices, catalysts and energy storage. Parameters such as film thickness, crystallinity, strain *etc*. determine the physical properties of thin films, such as electrical conductivity and transmittance. These properties directly affect the performance of practical devices. The atomic-scale structure of thin films can be single crystalline or polycrystalline, and these states have been used for device manufacturing. There have been many studies evaluating the crystalline structure of thin films using Rietveld refinement applied to grazing-incidence X-ray diffraction (GI-XRD) data, beginning with the first report by Quaas *et al.* (1998 ▸). Compared with conventional powder XRD (*i.e.* 2θ/θ measurements), GI-XRD enhances the thin film signal relative to the substrate signal by limiting the X-ray penetration depth near the critical angle. Several studies have reported that amorphous and partially crystalline thin films can be used in practical devices (Schropp *et al.*, 2007 ▸; Tsuruma *et al.*, 2021 ▸; Büschges *et al.*, 2025 ▸; Büschges *et al.*, 2026 ▸). In non-crystalline materials, device properties depend on short-range order (SRO) and medium-range order (MRO) structures rather than long-range order. Therefore, it is important to establish appropriate experimental methods to quantitatively evaluate SRO and MRO structures of thin films.

Pair distribution function (PDF) analysis enables the evaluation of the SRO and MRO of materials, and it has been widely applied to various research fields: batteries (Bréger *et al.*, 2007 ▸; Ohara *et al.*, 2016 ▸), ferroelectric materials (Petkov *et al.*, 2006 ▸; Yoneda & Kohara, 2009 ▸) and oxide glasses (Onodera *et al.*, 2020 ▸; Hashimoto *et al.*, 2022 ▸). It is necessary to measure total scattering data up to a sufficiently high momentum transfer, typically on the order of *Q* > 20 Å^−1^, because the maximum measurable momentum transfer *Q*_max_ determines the real-space resolution of peaks in PDF profiles (Peterson *et al.*, 2003 ▸). To obtain total scattering intensity with a high *Q*_max_ for thin film materials, several studies have reported thin film total scattering measurements performed using high-energy synchrotron radiation (Dippel *et al.*, 2019 ▸; Dippel *et al.*, 2020 ▸; Hoffman *et al.*, 2024 ▸; Zakutayev *et al.*, 2024 ▸). In the case of laboratory instruments, grazing-incidence total X-ray scattering (GI-TXS) techniques are necessary to obtain the structure factor *S*(*Q*) over a wide scattering vector magnitude (*Q*) range (Matsubara *et al.*, 1988 ▸; Bylin *et al.*, 2024 ▸). Bylin *et al.* have demonstrated GI-TXS measurement using a collimated incident X-ray beam and an absorption correction scheme based on a quasi-parallel beam approximation. With a parallel beam, typical incident angles in grazing-incidence geometry for high-energy X-rays are significantly smaller than 1°, leading to beam footprints on the sample that are larger by orders of magnitude. For example, the footprint size is approximately 380 times larger than the incident beam height when θ_in_ = 0.15°, and the brilliance of the incident beam is very low. A focused incident beam can solve this problem and enhance the incident flux on the sample to enable high-quality data collection, but it introduces a large convergence angle of the beam, which significantly affects the incident angle at laboratory sources. As a result, the incident angle can no longer be represented by a single value, which complicates the absorption correction in grazing-incidence geometry. Therefore, we propose a method for calculating the absorption factor of a film–substrate system by explicitly incorporating the non-linear dependence on the incident angle distribution.

In this paper, we present a GI-TXS measurement procedure and a data processing approach using a convergent incident beam to maximize the incident X-ray flux at the sample position. Furthermore, we estimate the substrate contribution to the observed total scattering intensity using the thickness, density and refractive index of thin film materials to correct for X-ray penetration effects of the convergent beam. We demonstrate GI-TXS measurements and obtain the PDF for an indium tin oxide (ITO) thin film deposited on a glass substrate to validate the present method.

## 2. Experimental

### 2.1. Theory

To apply GI-TXS to thin film samples, it is essential to account for X-ray behaviour in matter. The X-ray refractive index of a material, which is slightly less than 1, can be calculated using
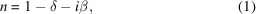
where δ and β are defined as follows:
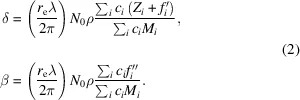

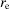
 is the classical electron radius (2.818 × 10^−15^ m), λ is the X-ray wavelength (Å), 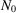 is Avogadro’s number (mol^−1^), ρ is the mass density (g cm^−3^), 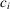 is the molar fraction of the *i*th atom, 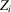 is the atomic number of the *i*th atom, 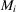 is the atomic mass of the *i*th atom, and 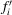 and 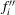 are the anomalous dispersion terms of the atomic scattering factor of the *i*th atom. As shown in equation (1), the X-ray refractive index is a complex number. Here, δ and β mainly represent the refraction and absorption effects of X-rays in matter, respectively.

For a parallel beam, the absorption factor for a thin film, including refraction effects in matter (Matsubara *et al.*, 1988 ▸), is given by
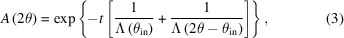
where *t* is the thickness of the thin film, θ_in_ is the incident angle to the thin film and Λ is the X-ray penetration depth (Omote, 2002 ▸):
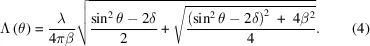
For a convergent beam focused at the sample position, the thin film is illuminated by a range of incident angles within 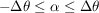 (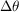 is half of the convergence angle of the optics). Therefore, the absorption factor depends not only on refraction in matter but also on the convergence angle distribution, as follows:
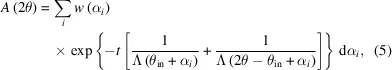
where 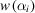 is the weight factor for the convergent beam, 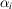 is the convergence angle within 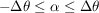 and 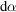 is the bin size. In this study, 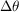 and 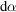 are 0.14° and 0.001°, respectively. 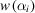 is defined as
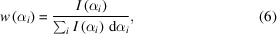
where 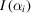 is the beam profile intensity at 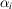.

Finally, we obtain the total scattering intensity of the thin film 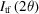 from the following equation:
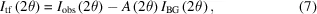
where 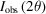 is the total scattering intensity from both the thin film and substrate, and 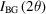 is the total scattering intensity contributed only by the substrate. The normalized intensity 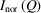 is obtained from 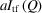, where *a* is a scaling coefficient determined using the Krogh-Moe–Norman formula (Krogh-Moe, 1956 ▸; Norman, 1957 ▸):
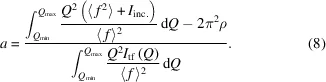


This normalization ensures that the resulting structure factor satisfies the correct asymptotic behaviour at high *Q* and is consistent with the physical normalization condition. Here, 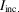 is the Compton scattering intensity referenced from tabulated values, ρ is the number density of the sample, and 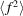 and 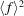 are defined as follows (Egami & Billinge, 2012 ▸; Bylin *et al.*, 2024 ▸):
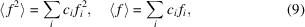
where 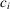 and 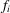 are the molar concentration and atomic scattering factor of the *i*th element, respectively.

*Q* is the magnitude of the scattering vector, defined as
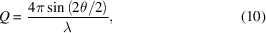
where 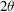 is the scattering angle and λ is the X-ray wavelength. The structure factor 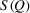 is then obtained from 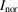 as follows:
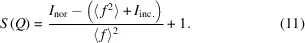
The PDF [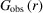] is obtained by Fourier transforming 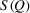:
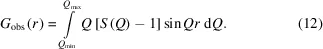


### 2.2. Total scattering and X-ray reflectivity measurements

GI-TXS measurement was performed using a SmartLab high-resolution X-ray diffractometer (Rigaku Corporation) equipped with a HyPix-3000HE detector (Rigaku Corporation), which is optimized for high-energy X-ray detection. Incident Ag *K*α (λ = 0.5608 Å) X-rays were monochromated by an elliptic *d*-space graded multilayer mirror focused on the sample position (Rigaku Innovative Technologies). The incident X-ray distribution of the convergent beam was characterized with a Si(12 0 0) crystal at the sample position as shown in Fig. 1 ▸. The FWHM is about 0.19°. An ITO thin film (GEOMATEC Co., Ltd), whose thickness is designed to be 90 nm on a glass substrate, was prepared as a sample. Total scattering data were collected at a grazing-incidence angle of 0.14° over a scattering angle range of 2.0° ≤ 2θ ≤ 140.0°, corresponding to *Q*_min_ = 0.39 Å^−1^ and *Q*_max_ = 21.06 Å^−1^. The angular step size was 0.05°, with a counting time of 15 s per step. The thin film total scattering profile is extracted by applying equations (4)–(7), and other intensity corrections are performed using established procedures (Thijsse, 1984 ▸).

To evaluate the film parameters of ITO, an X-ray reflectivity measurement was also performed on a SmartLab. A parallel Cu *K*α_1_ (λ = 1.5405 Å) beam was obtained using a parabolic *d*-space graded multilayer mirror and a Ge(220) double-crystal monochromator. The ITO thin film density, thickness and roughness were determined by X-ray reflectivity (XRR) analysis (plugin in *SmartLab Studio II* software, Rigaku Corporation).

## 3. Results and discussion

### 3.1. GI-TXS measurement for SiO_2_ glass plate

To validate the GI-TXS measurement, an SiO_2_ glass plate is one of the simplest samples because it does not require an absorption factor to subtract the background profile (*i.e.* air scattering). Fig. 2 ▸ shows a comparison of *S*(*Q*) obtained from GI-TXS and Debye–Scherrer geometry using an SiO_2_ glass plate with a thickness of 1 mm and an SiO_2_ glass rod with a diameter of 0.5 mm as a sample. The *S*(*Q*) from GI-TXS is consistent with that from Debye–Scherrer geometry over a wide *Q* range. This result indicates that GI-TXS measurements can be applied to evaluate the local structure in thin films.

### 3.2. ITO thin film analysis

Fig. 3 ▸ shows the observed and calculated XRR profile after refinement. The corresponding refinement results indicate that the ITO thin film can be described as a single layer with a density of 6.71 g cm^−3^, a film thickness of 76.6 nm and a surface roughness of 2.0 nm, as summarized in Table 1 ▸. These refined parameters were subsequently used to calculate the absorption factor for correcting the substrate contribution.

Fig. 4 ▸ compares the extracted total scattering intensity of the thin film obtained using two different absorption correction schemes. The convergent beam correction incorporates the experimentally determined incident X-ray beam distribution shown in Fig. 1 ▸, whereas the conventional parallel beam correction assumes a single incident angle (θ_in_ = 0.14°), as commonly adopted in previous studies and shown here as a reference. The discrepancy between the two results suggests that the single-angle approximation is insufficient under the present experimental conditions, where the incident angle distribution is broad due to beam convergence. The observed intensity *I*_obs_ indicates a broad peak related to the glass substrate at around 2θ = 10°. The absorption correction taking into consideration the convergent beam effect successfully extracts the ITO thin film intensity *I*_tf_ from *I*_obs_. On the other hand, the absorption correction for the parallel beam fails to extract the true film signal, as *I*_tf_ still contains a broad peak originating from the glass substrate. Fig. 5 ▸ shows the observed data after polarization correction, normalized to the atomic scattering factor. *I*_tf_ is consistent with the atomic scattering factor over the entire *Q* range. These results indicate that an absorption correction is required to consider the contribution from the beam profile at each convergence angle.

Fig. 6 ▸ shows a comparison of *S*(*Q*) for the ITO thin film and powder In_2_O_3_. In the high-*Q* region (*Q* > 8 Å^−1^), the frequency and magnitude of the oscillations of the ITO thin film, which are related to the local structure of ITO, are consistent with those of powder In_2_O_3_. In contrast, the intensity ratio of reflections in the low-*Q* region 2 < *Q* < 4 Å^−1^ is quite different from that of powder In_2_O_3_. This suggests that ITO forms an oriented thin film. Rietveld analysis was performed on GI-TXS profiles of the ITO thin film. The observed GI-TXS profile is well reproduced by the calculated profile obtained from Rietveld refinement, as shown in Fig. S1 in the supporting information. The preferred orientation was quantified using the March–Dollase function, with the [001] direction selected as the preferred orientation vector based on the ITO(004) reflection. The refined March–Dollase parameter of 0.3931 (12), which is significantly smaller than unity, is characteristic of the plate-like crystallites and indicates a preferred orientation along the *c*-axis direction.

The obtained *G*(*r*) of the ITO thin film is different from that of powder In_2_O_3_, as shown in Fig. 7 ▸. Since the intensity ratio of the reflections differs from that of the powder In_2_O_3_, we conclude that the ITO forms an oriented thin film on the glass substrate. To estimate the partial correlation contributions, the Faber–Ziman weighting factor (Faber & Ziman, 1965 ▸) of ITO for X-ray*G*(*r*) is assumed below:
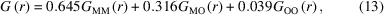
where M and O denote metal and oxygen, respectively. The Faber–Ziman weighting factors are determined by both atomic concentrations and the atomic numbers in the X-ray*G*(*r*). Since the Sn concentration is much lower than that of In in typical ITO, the contribution of Sn-related partial correlations to the total *G*(*r*) is relatively small. Therefore, as a practical approximation, In and Sn partial correlations are treated as a single metal correlation. The equation indicates that M–M and M–O partial correlations mainly contribute to the observed *G*(*r*). At *r* < 4 Å, the peak positions of the ITO thin film are close to those of powder In_2_O_3_. In contrast, the *G*(*r*) peak shapes of the ITO thin film show different features compared with those of powder In_2_O_3_ in the region of *r* > 6 Å. These tendencies are consistent with previous reports on the comparison of *G*(*r*) between textured thin film materials and the homogeneous state (Dippel *et al.*, 2019 ▸; Roelsgaard *et al.*, 2019 ▸; Hoffman *et al.*, 2024 ▸). Therefore, the observed differences in the peak intensities are interpreted as being consistent with the presence of preferred orientation in the ITO thin film. A more detailed study of the texture effect in *G*(*r*) is left for future work.

## 4. Conclusion

In this study, we propose a GI-TXS measurement protocol and data analysis process using a convergent beam at the sample position. The thin film *S*(*Q*) can be obtained by correcting reflection and absorption effects using the fundamental physical parameters of the thin film and the X-ray refractive indices. The proposed method enables reliable measurements of thin film *S*(*Q*) up to *Q*_max_ = 21 Å^−1^ using a laboratory diffractometer. The improvement arises from the non-linear absorption factor that accounts for the incident angle distribution of the convergent beam, thereby overcoming the limitations of the conventional single-angle approximation in the grazing-incidence geometry. It is confirmed that the obtained *S*(*Q*) of the ITO thin film in the high-*Q* region agrees well with that of the powder In_2_O_3_ sample in both the frequency and amplitude of the oscillations. This is reasonable because the short-range structure is expected to be similar in both the thin film and powder states, and the oscillations in this region reflect the short-range structure. However, it should be noted that, in the present out-of-plane total X-ray scattering measurements, the scattering profile contains contributions from different reciprocal-lattice vectors (**G** vector) for the incident and scattered X-rays at each 2θ position. Therefore, the resulting *S*(*Q*) represents a mixture of different **Q**-vector directions. In the future, we will attempt to measure the total scattering profile along the same **Q** vector via in-plane measurements. Despite this limitation, the present GI-TXS method can be widely applied to evaluate the SRO and MRO structures of amorphous and partially crystalline thin films.

## Supplementary Material

Supporting information: Fig. S1 which shows the Rietveld refinement results. DOI: 10.1107/S1600576726007181/jur5014sup1.pdf

## Figures and Tables

**Figure 1:**
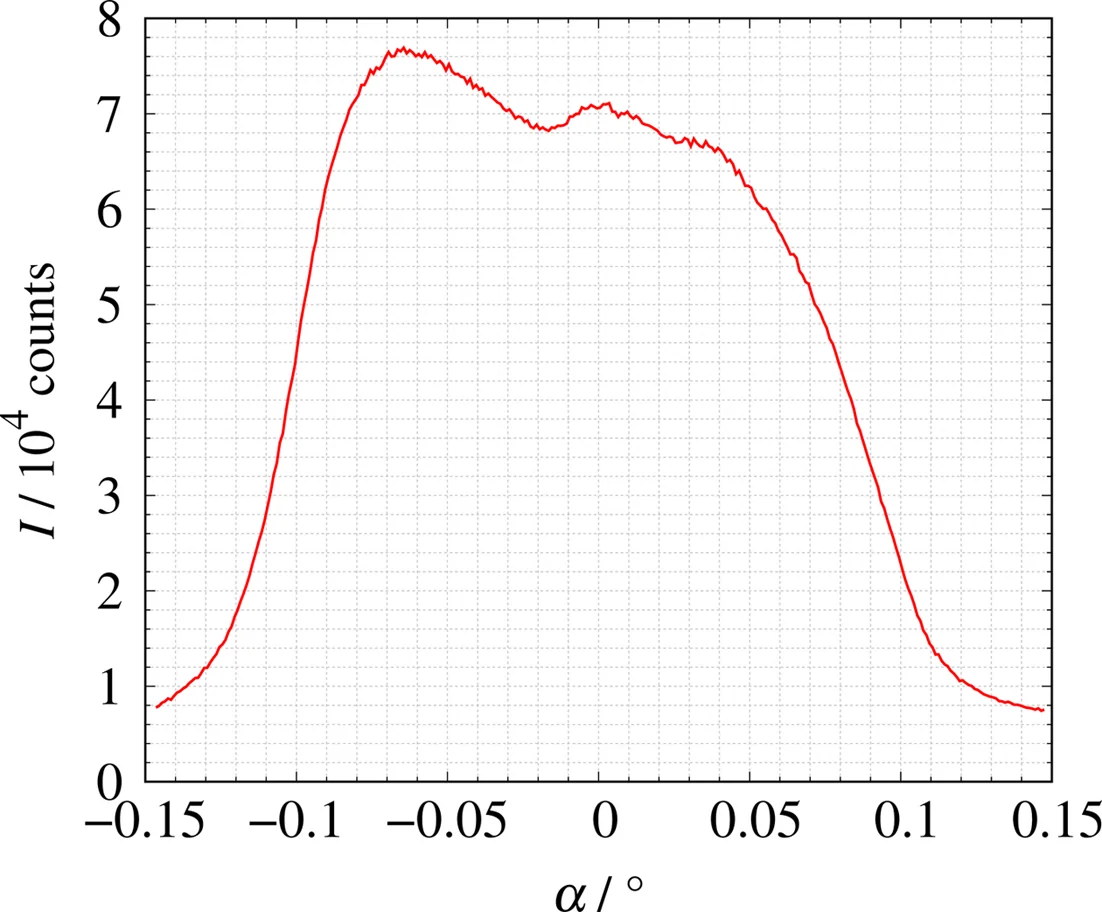
Convergent beam profile characterized with a Si(12 0 0) crystal (*i.e.* ω = 38.173°) at the sample position.

**Figure 2:**
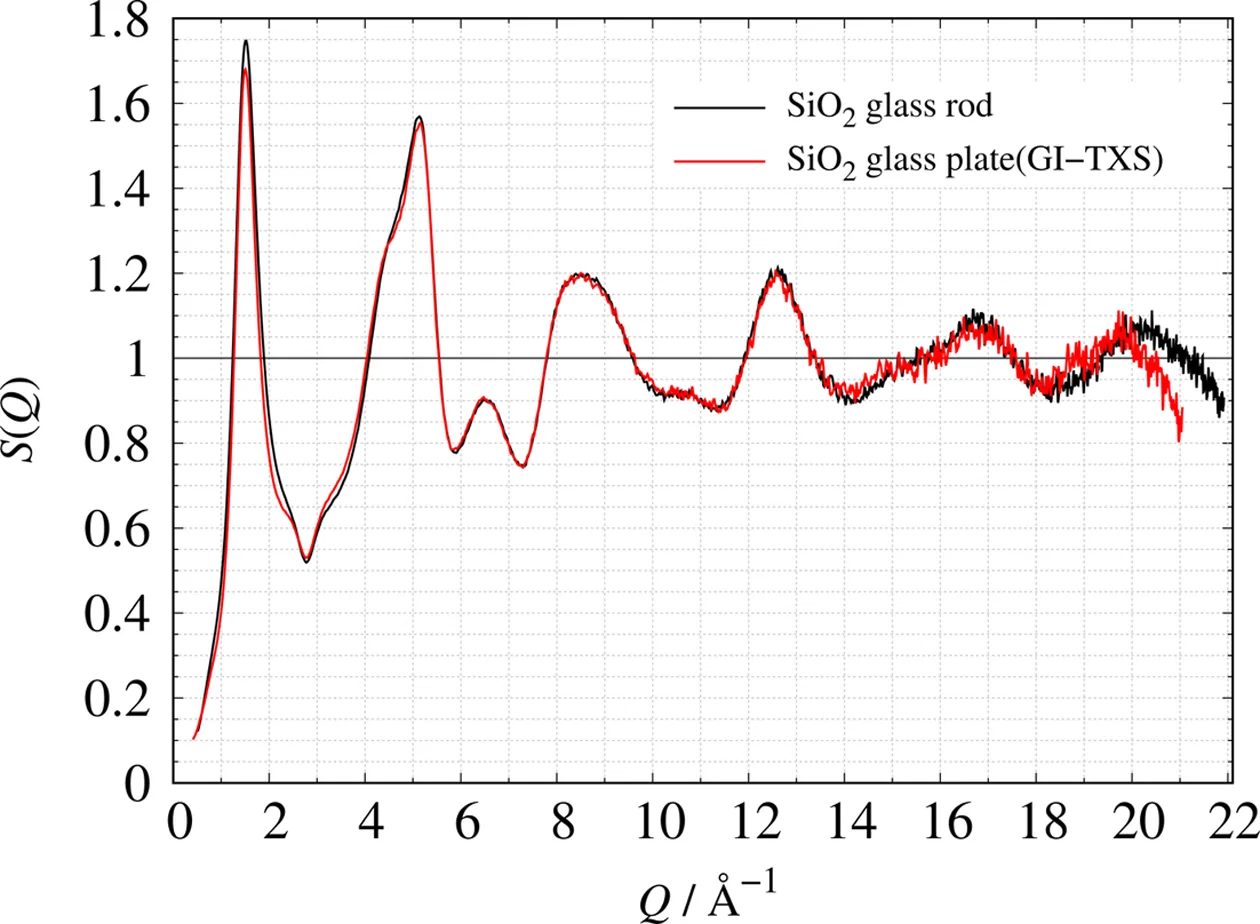
Comparison of SiO_2_ glass *S*(*Q*) obtained from GI-TXS (red) and Debye–Scherrer geometry (black).

**Figure 3:**
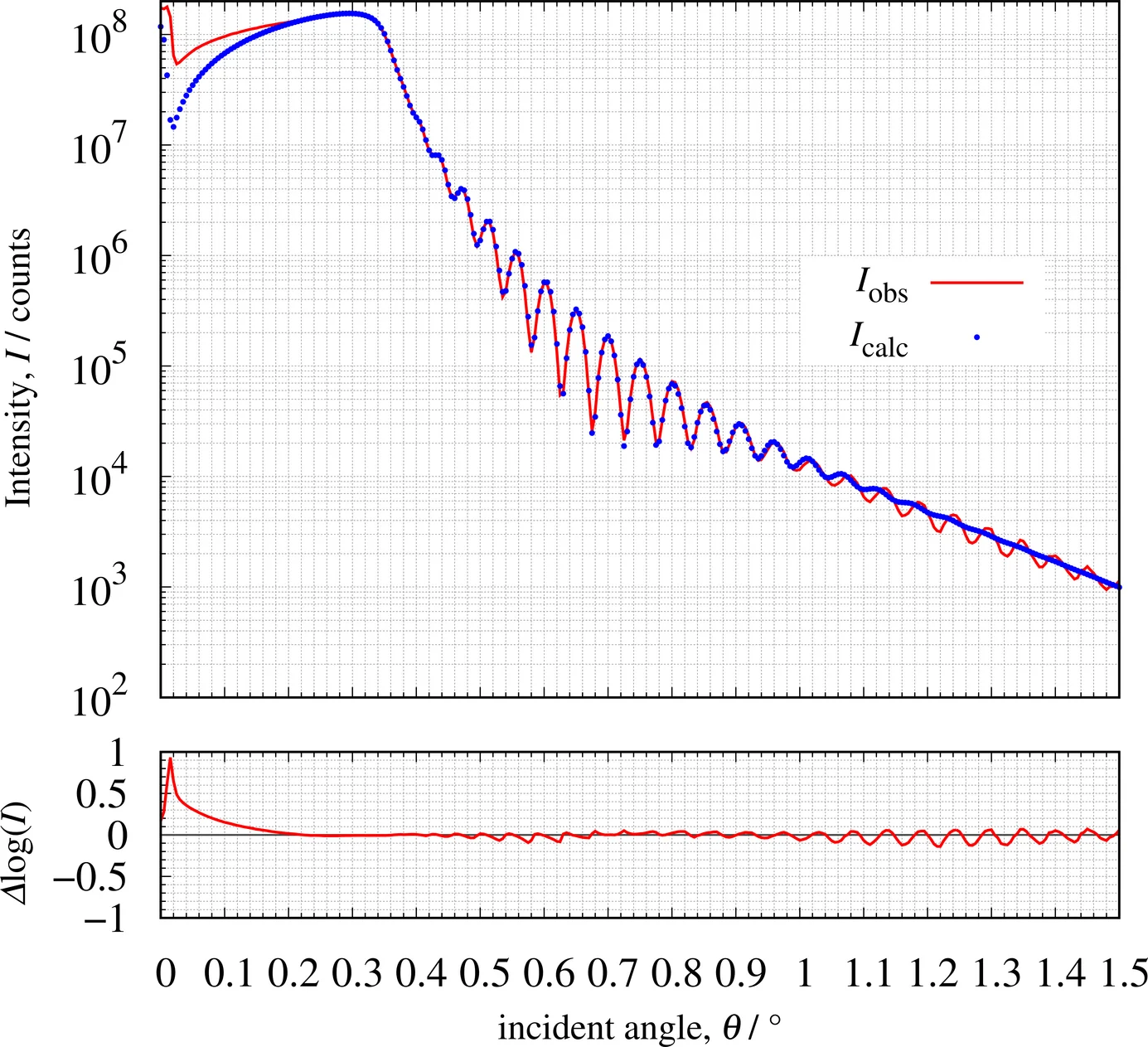
Comparison of X-ray reflectivity profiles for the ITO thin film (top) and corresponding deviation curve (bottom). The red solid line shows the observed intensity. Blue filled circles show the calculated profile obtained using the refined ITO thin film parameters. The reliability factor *R* = 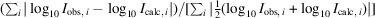 is 0.74% after refinement.

**Figure 4:**
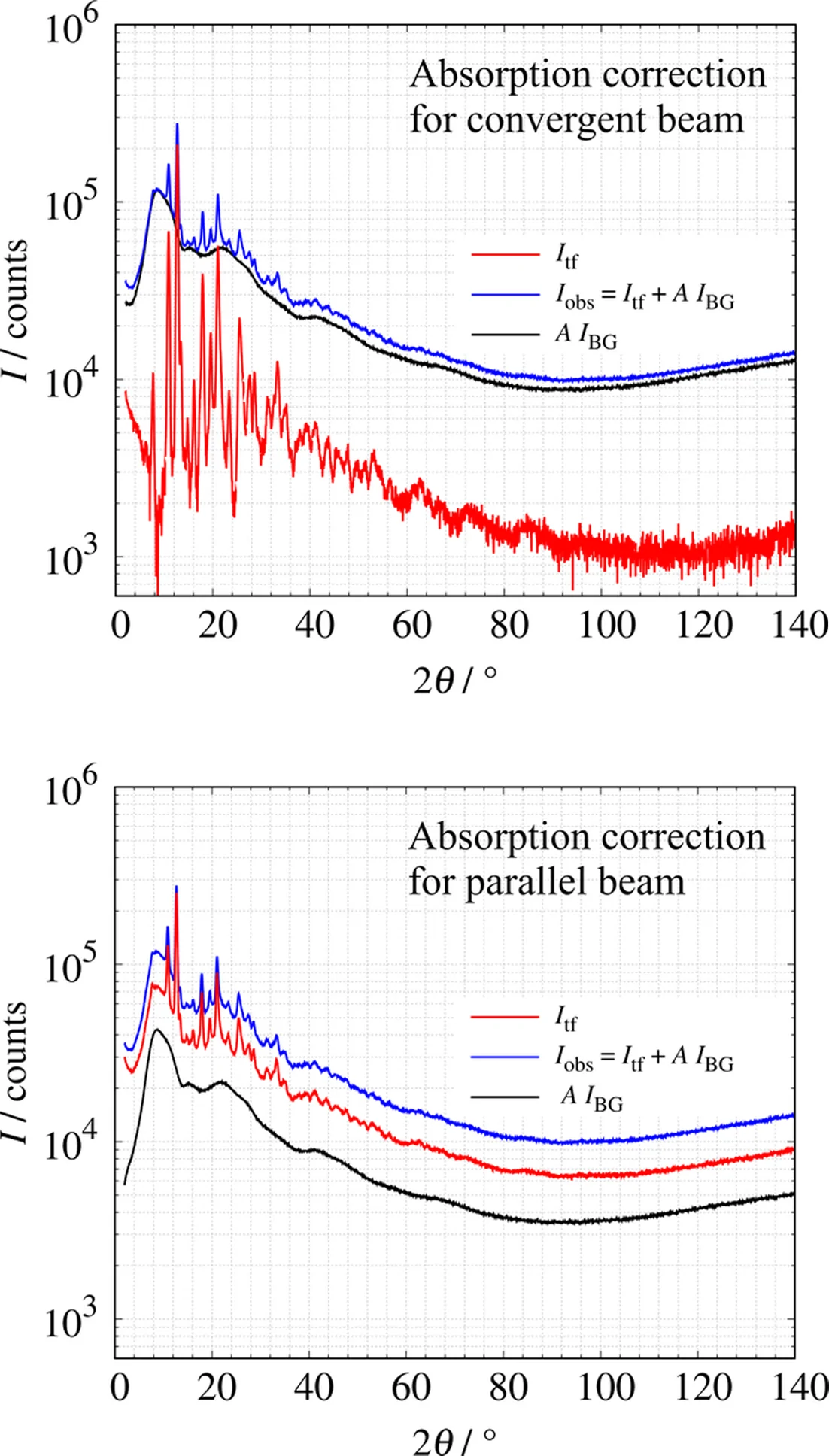
Comparison of absorption correction results: (top) absorption correction for a convergent beam using equations (5) and (6); (bottom) absorption correction for a parallel beam using equation (3). The penetration depth of the ITO thin film was calculated from the thin film parameters obtained by XRR analysis.

**Figure 5:**
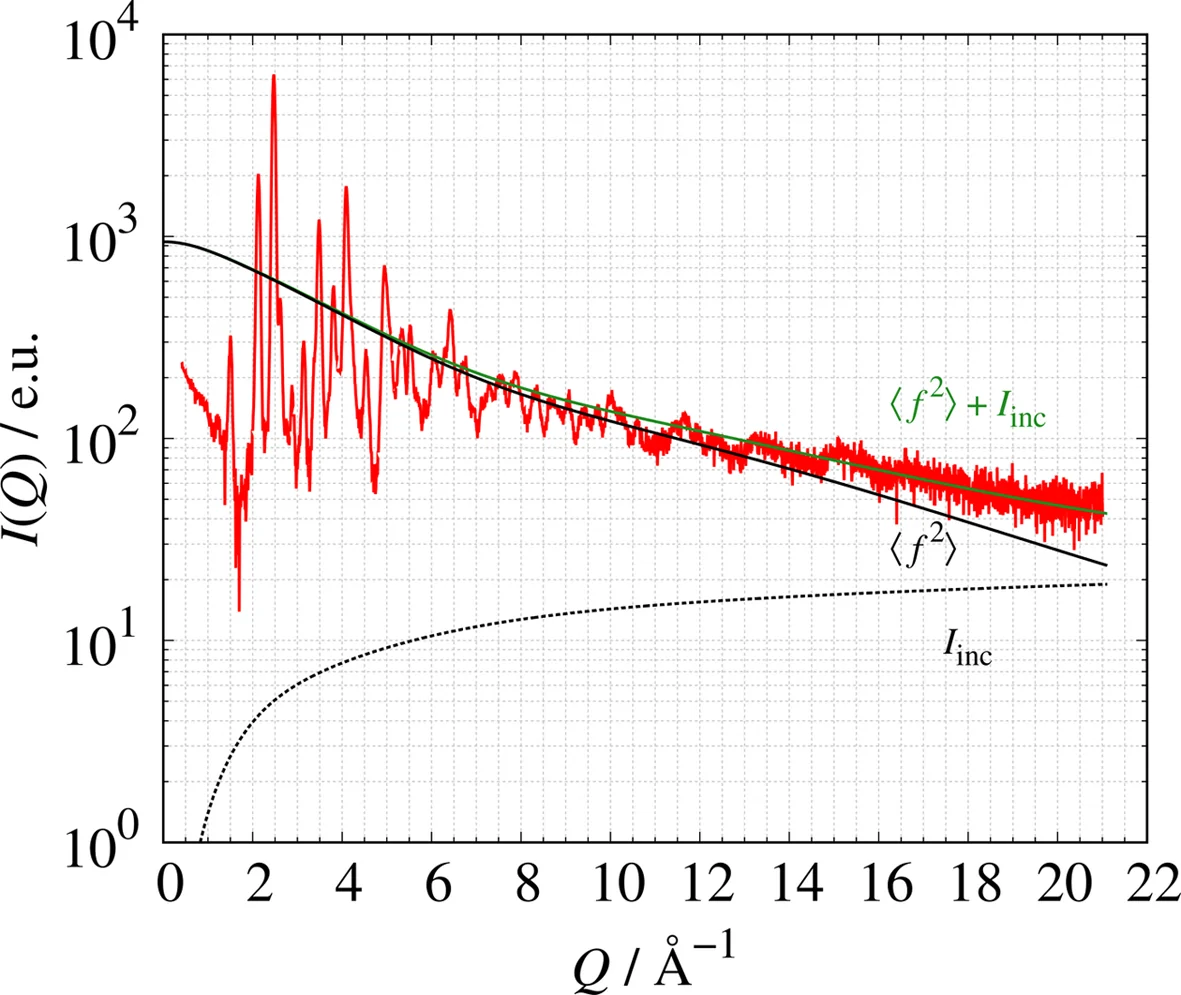
Total scattering intensity of the ITO thin film normalized by the sum profile of the atomic scattering factor 〈 *f* ^2^〉 and the Compton scattering intensity *I*_inc_. Red line: total scattering intensity of the ITO thin film; green line: sum profile of 〈 *f* ^2^〉 and *I*_inc_; black solid line: 〈 *f* ^2^〉; black dashed line: *I*_inc_.

**Figure 6:**
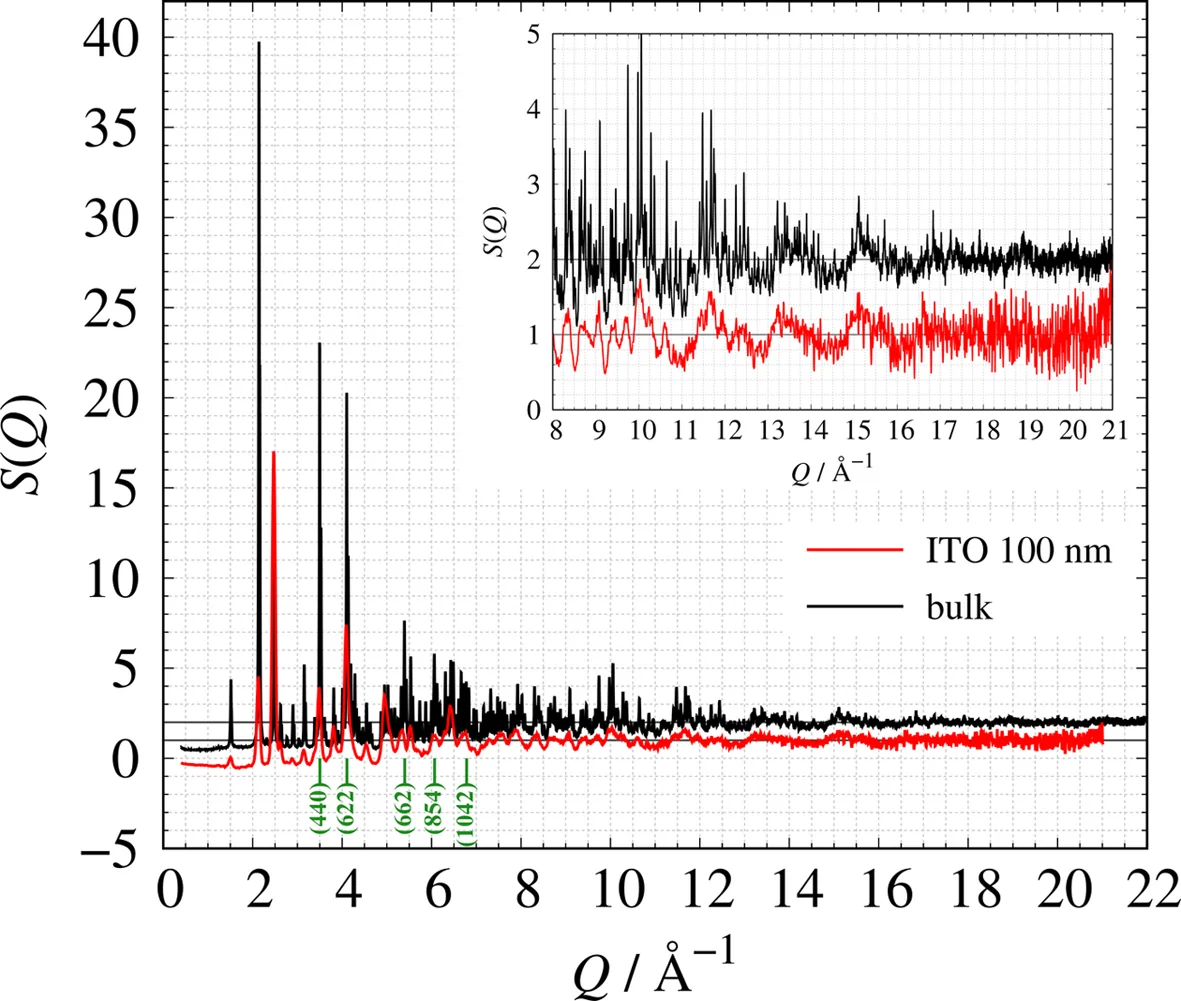
Comparison of *S*(*Q*) for the ITO thin film (red) and powder In_2_O_3_ (black). The inset also shows a zoomed-in view over 8 < *Q* < 21 Å^−1^. For clarity, an offset has been applied to the *S*(*Q*) of powder In_2_O_3_.

**Figure 7:**
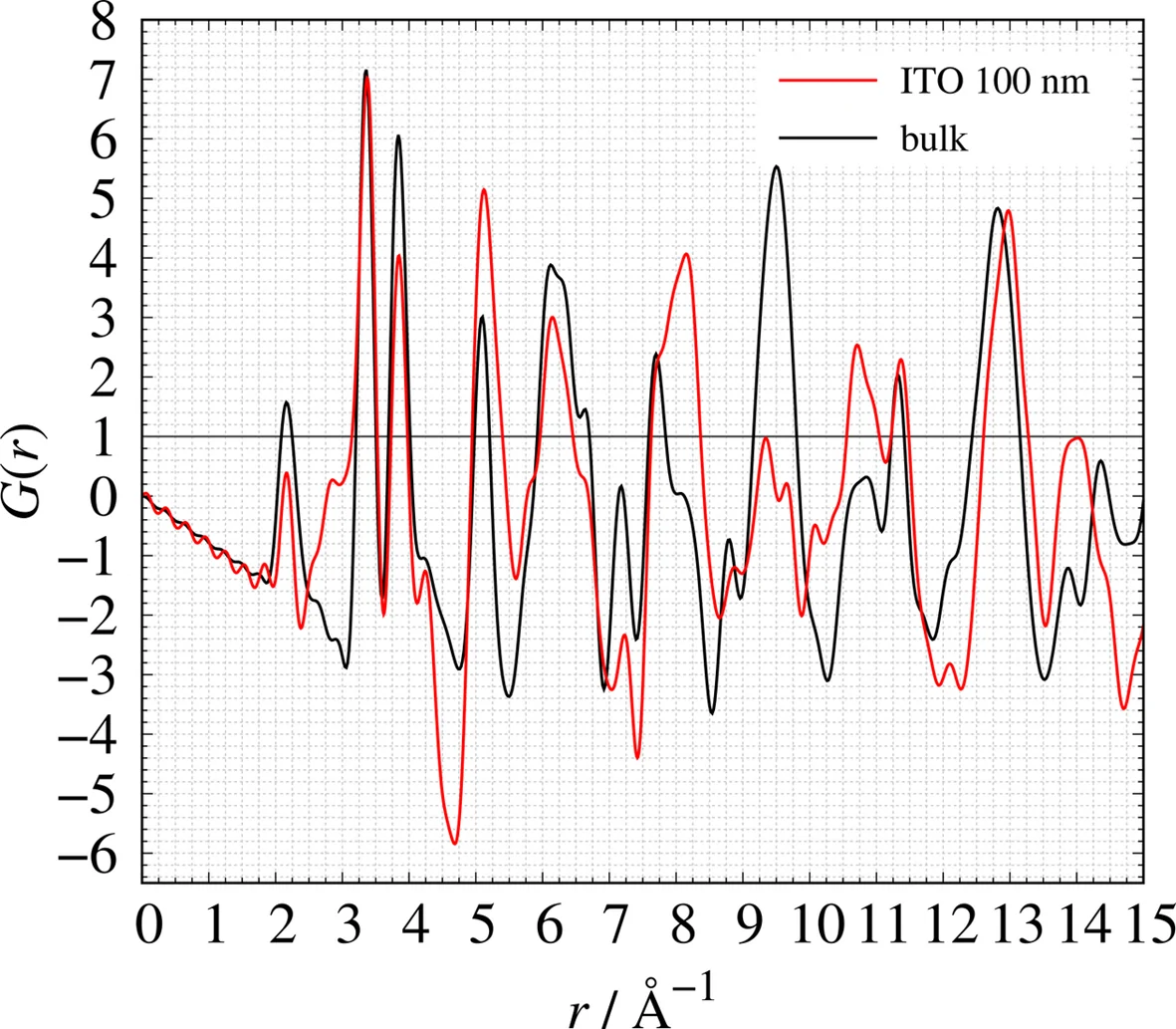
Comparison of *G*(*r*) between ITO thin film (red) and powder In_2_O_3_ (black).

**Table 1:** ITO thin film parameters after XRR analysis

Layer No.	Material	Density (g cm^−3^)	Thin film thickness (nm)	Film roughness (nm)
0 (substrate)	Glass	2.21	–	0.73
1	ITO	6.71	76.6	2.03

## Data Availability

The data are available upon request.
