# Supplementary material for: Probing local atomic structure in thin films by grazing-incidence total X-ray scattering with a convergent X-ray beam
Source: J Appl Crystallogr. 2026 Aug 24;59(Pt 5):1420–5. doi: 10.1107/S1600576726007181 (PMC13636040; doi:10.1107/S1600576726007181)
Supplement: Supplementary file 1 [file j-59-01420-sup1.pdf]

JOURNAL OF
APPLIED
CRYSTALLOGRAPHY

Volume 59 (2026)

Supporting information for article:

Probing Local Atomic Structure in Thin Films by Grazing-Incidence Total X-ray Scattering with a Convergent X-ray Beam

Masatsugu Yoshimoto, Kazuki Ito and Kazuhiko Omote

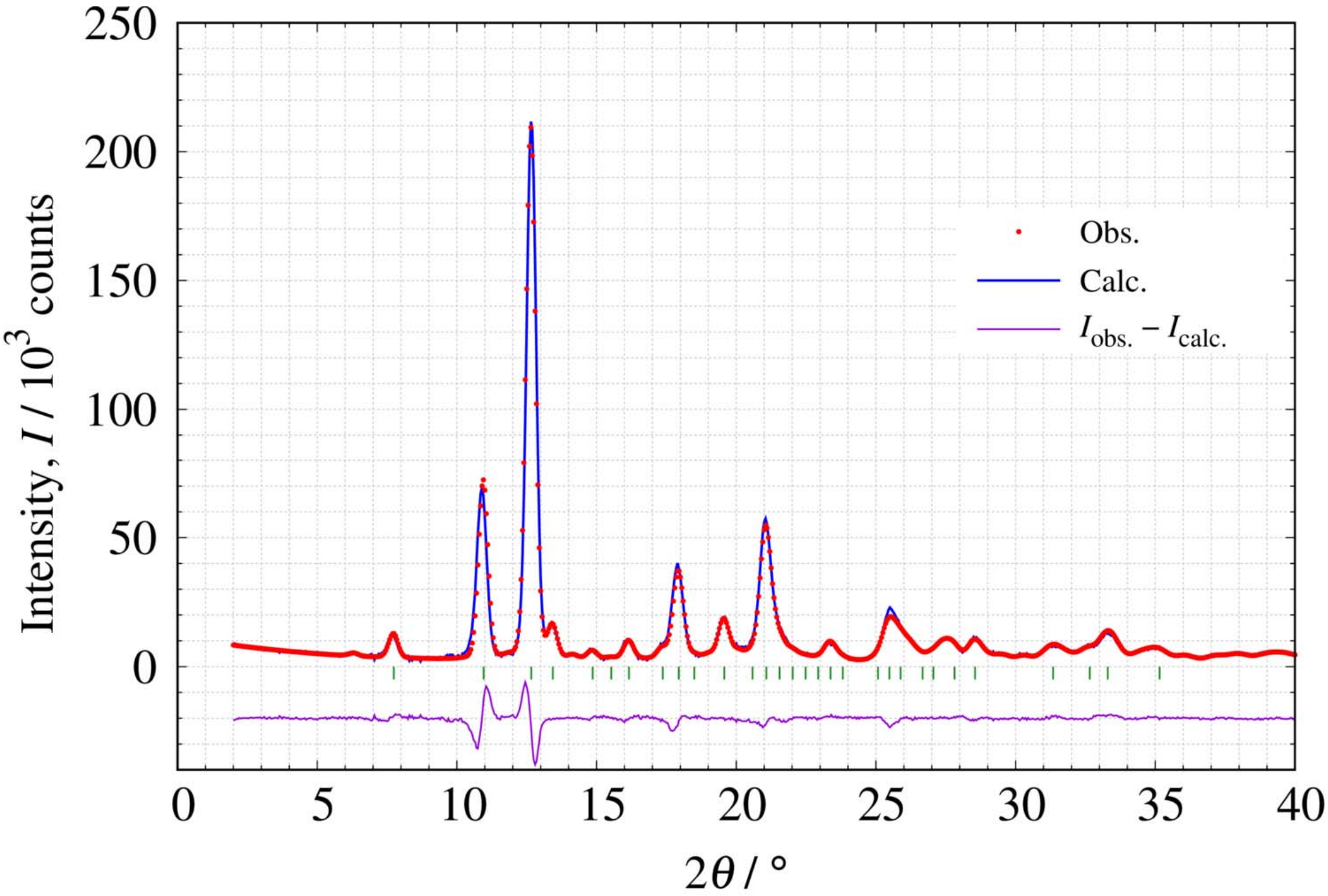

Figure S1 Comparison of observed GI-TXS profile of ITO thin film (red filled circle) and the calculated profile (blue solid line) obtained by Rietveld refinement using PowderXRD plugin of SmartLab studio II. The corresponding differences (violet solid line), and Miller index (green solid line) of ITO were also shown in this figure. The refinement results (R_{wp} = 10.67%, R_p = 7.79%, R_e = 1.52% and S = 7.00) indicates good agreement between the observed and calculated profiles. The refinement enables reliable determination of the structural parameter and the preferred orientation of ITO thin film.
